# Receiving threatening or obscene messages from a partner and mental health, self-harm and suicidality: results from the Adult Psychiatric Morbidity Survey

**DOI:** 10.1007/s00127-021-02113-w

**Published:** 2021-07-28

**Authors:** Sally McManus, Paul E. Bebbington, Leonie Tanczer, Sara Scott, Louise M. Howard

**Affiliations:** 1 Violence and Society Centre, City, University of London, London, UK; 2 National Centre for Social Research, 35 Northampton Square, London, EC1V 0AX UK; 3 Psychiatry Division, University College London, London, UK; 4 Faculty of Engineering Science, University College London, London, UK; 5 DMSS, North Dalton, England, UK; 6 Section of Women’s Mental Health, Institute of Psychology, Psychiatry and Neuroscience, Kings College London, London, UK

**Keywords:** Intimate partner violence, Emotional abuse, Cyber bullying, Threat, Mental health, Self-harm, Suicidality

## Abstract

**Purpose:**

Threatening or obscene messaging is repeated, unwanted texts, emails, letters or cards experienced by the recipient as threatening or obscene, and causing fear, alarm or distress. It is rarely examined as an aspect of intimate partner violence. We describe the prevalence of exposure to threatening/obscene messaging from a current or ex-partner; characteristics of victims; and associations with other forms of violence and abuse, mental disorder, self-harm, and suicidality.

**Methods:**

Cross-sectional probability-sample survey of the general population in England aged 16 + . Multivariable regression modelling tested associations between receipt of threatening/obscene messaging and current common mental disorder, past-year self-harm and suicidality.

**Results:**

Threatening/obscene messages were received from a current/ex-partner by 6.6% (95%CI: 5.9–7.3) of adults who had been in a relationship; 1.7% received these in the past year. Victims were more likely to be female, under 35, single or divorced, socioeconomically disadvantaged, and to have experienced other forms of sexual and partner violence and abuse. Those who received threatening/obscene messages in the past year were more likely to experience common mental disorder (adjusted odds ratio 1.89; 1.01–3.55), self-harm (2.31; 1.00–5.33), and suicidal thoughts (2.00; 1.06–3.78).

**Conclusion:**

Threatening/obscene messaging commonly occurs in the context of intimate partner violence. While often occurring alongside sexual and physical violence, messaging has an additional association with mental disorder and suicidality. Routine enquiry in service settings concerning safety, including those working with people who have escaped domestic violence, should ask about ongoing contact from previous as well as current partners. This should include asking about messaging, as well as other forms of potentially technology-enabled abuse which may become increasingly common.

**Supplementary Information:**

The online version contains supplementary material available at 10.1007/s00127-021-02113-w.

## Introduction

Violence, abuse, and bullying are widely recognised as factors contributing to both the onset and continuation of mental disorder [1, 2]. England’s Department of Health and Social Care (DHSC) recommends that healthcare professionals ask patients about current and historic exposure to violence and abuse, to identify risk factors, establish safety, and support recovery [3–5]. A dose–response relationship has emerged with the risk of poor mental health being greater where experience of violence and abuse has been more pervasive [6]. People exposed to one type of violence and abuse are more likely to also experience other types, at the same time or subsequently [7].

One of the most common forms of violence in adulthood is that from an intimate partner (IPV). The World Health Organisation lists different types of IPV, including physical violence, sexual violence, emotional or psychological abuse, and controlling behaviours, involving isolation, monitoring, and restricted access to resources [8]. In the UK, the *Serious Crime Act 2015* was the first to recognise controlling or coercive behaviour in the context of an intimate or family relationship as an offence [9], and the *Domestic Abuse Act 2021* specifically recognises economic and technology-related abuse [10, 11]. However, a tension has been noted between such public policy and the gendered reality of domestic violent crime [12].

Emotional abuse is known to predict poor mental health as strongly as physical or sexual abuse [13] and includes insults, belittling, intimidation and threats, delivered face to face or communicated in other ways. The *Malicious Communications Act 1988* had made it illegal in England and Wales to send or deliver letters or other articles for the purpose of causing distress or anxiety, and in 2001 the legislation was updated to also apply to electronic communications [14]. From March 2019 to March 2020, 36% of all domestic violence-related stalking and harassment offences recorded by police in England and Wales were malicious communications [15]. Police forces, however, have been found to often not record—or to mis-record—such reports [16]. Crime statistics therefore significantly underestimate the extent of abusive behaviours conducted through communications, including digital communications, in the population, with further changes to likelihood of reporting occurring in the context of COVID-19 restrictions [17].

Global developments in communication technology have the potential to enable malicious communications to become increasingly pervasive, immediate, and intrusive [18]. While the UK government’s recent *Online Harms* white paper acknowledged this, it made no reference to its occurrence in the context of IPV nor accounted for the growing risk from Internet-connected devices [19]. Instead attention has focussed on threatening or obscene messaging in other situations: such as hate speech [20], children’s online behaviour [21], and conduct on social media. Furthermore, research on sexting [22] and cyberbullying has tended to focus on peer-related bullying rather than IPV. Malicious communications can form an aspect of technology-facilitated abuse (‘tech abuse’), which includes the monitoring of victims through networked camera systems, stalking via software and tracking tools frequently referred to as “stalkerware” [23, 24], and other forms of “smart” technological systems such as Internet-connected household appliances to coerce, control, and harm others [25]. Limited recognition has been given to these newer forms of abuse, especially in the context of emotional abuse and coercive control from a current or previous partner. In 2020, the UK Law Commission [26] launched a consultation on reforming the Malicious Communications Act 1988 and Communications Act 2003, in light of developments in online communication and recognising the inherently gendered nature of online abuse and its potential for causing harm.

While England’s mental health survey did not ask specifically about tech abuse, it did include questions on receipt of repeated threatening and obscene messages from a current or previous partner, spanning both technology-facilitated and other forms of communication. The aims of this study were to establish:

- The first prevalence estimates of exposure to threatening/obscene messaging from current or previous partners among adults in England,
- The characteristics of people exposed to such messages at any point in adulthood, and specifically within the past year,
- Whether past-year exposure to such messaging was associated with common mental disorder, non-suicidal self-harm, suicidal thoughts, and suicide attempt, after adjustment for demographic and socioeconomic circumstances and experience of other forms of violence and abuse.

## Methods

We carried out a secondary analysis of data from the 2014 Adult Psychiatric Morbidity Survey (APMS), a general population survey of the mental health of adults resident in private households in England. A stratified random probability sampling design was used, based on the selection of addresses from the Postcode Address File (PAF). This involved multiple stages: sampling primary sampling units (PSUs); addresses within selected PSUs; and one individual aged 16 or over from each selected address. People living in communal or institutional establishments, in temporary housing, or sleeping rough, were not in scope. Fieldwork took place May 2014–September 2015, with verbal informed consent.

Seven thousand five hundred fourty-six participants were interviewed, a response rate of 57%. Weights were developed to take account of selection probabilities and non-response, in order to render results representative of the household population. Interviews averaged 1.5 h and were conducted in people’s own homes (or elsewhere, if preferred) by trained research interviewers. The questionnaire was largely administered face-to-face using computer-assisted interviewing. Some information considered more sensitive was collected by self-completion for increased privacy, with participants keying their responses directly into a laptop. Interviewers assisted respondents with literacy or eyesight difficulties. 7058 participants carried out the self-completion component: factors associated with declining the self-completion included being male and being older [27]. Further methodological details are published elsewhere [28, 29].

## Measures

### Exposure

The receipt of repeated threatening or obscene messages was established by asking: ‘Has a partner or ex-partner ever sent you more than one unwanted letter, email, text message or card that was either obscene or threatening and which caused you fear, alarm or distress?’ On- and offline messaging could not be disaggregated, nor whether messages were experienced as either threatening or obscene. The question was administered by self-completion as part of a module about violence and abuse from a partner or ex-partner. It was only asked of those who had had a partner (boyfriend/girlfriend/spouse) at some point. Follow-up questions established how recently the abuse occurred. If the experience had happened in the past 12 months, respondents were asked about frequency.

### Outcomes

Common mental disorders (CMDs) were assessed using the Clinical Interview Schedule-Revised (CIS-R). This is an interviewer-administered structured interview covering the presence of non-psychotic symptoms in the week prior to interview. It can provide prevalence estimates for six CMDs according to ICD-10 clinical criteria [30]: generalised anxiety disorder, phobia, obsessive compulsive disorder, panic disorder, depression and other common mental disorder not otherwise specified [31]. The outcome variable was binary, indicating either presence or absence of any CMD.

Suicidal thoughts: In the face-to-face section of the interview, participants were asked: ‘Have you ever thought of taking your life, even though you would not actually do it?’ An affirmative response was followed with a question about when this had last occurred, and a variable was derived indicating those reporting such thoughts in the past year.

Non-suicidal self-injury and suicide attempt: the 5th Diagnostic and Statistical Manual of Mental Disorders includes non-suicidal self-injury and suicidal behaviour disorder as conditions for further study [32]. Suicide attempts and non-suicidal self-harm were examined separately. Questions about suicide attempts within the past year were asked in both the face-to-face and self-completion sections of the interview: ‘Have you ever made an attempt to take your life, by taking an overdose of tablets or in some other way?’ A derived variable combined reports of a suicide attempt in the past year in either section of the interview. Non-suicidal self-harm was also asked both face-to-face and in the self-completion section: ‘Have you ever deliberately harmed yourself in any way but not with the intention of killing yourself?’ Non-suicidal self-harm in the past year similarly drew on reports from either the face-to-face or self-completion section. While agreement was high, rates from the self-completion section were higher [29].

Harmful or dependent alcohol use: was measured using the Alcohol Use Disorders Identification Test (AUDIT) [33]. The AUDIT takes the year before the interview as a reference period, consists of 10 items and covers alcohol consumption, alcohol-related harm, symptoms of alcohol dependence. Answers to all questions were scored from zero to four, and summed to give a total score ranging from 0 to 40. A binary indicator was produced for scores of 1–15 and 16 or over, with the latter indicative of a level of alcohol use considered harmful drinking and/or dependent.

### Covariates

Emotional, physical and sexual violence and abuse in childhood and adulthood were asked about in the self-completion section of the interview. Those relating to violence by a current or former partner were adapted from questions in the British Crime Survey, originally based on the Conflict Tactics Scale (CTS) [34]. Emotional abuse in childhood was indicated by an affirmative response to: ‘Before you were 18, did you get scared or feel really bad because an adult in your life called you names, said mean things to you, or said they didn’t want you?’ Physical violence in childhood was based on: ‘Not including smacking, before you were 18, did an adult in your life hit, beat, kick, or physically hurt you in any way?’ Childhood sexual abuse was derived from two questions relating to non-consensual sexual contact and forced sexual intercourse before age 16. Emotional abuse from a current or former partner was based on an affirmative response to: ‘Has a partner or ex-partner ever repeatedly belittled you to the extent that you felt worthless?’ Physical violence from a partner was established by asking: ‘Has a partner or ex-partner ever pushed you, held or pinned you down or slapped you?’ and ‘Has a partner or ex-partner ever kicked you, bit you, or hit you with a fist or something else, or threw something at you that hurt you?’ Sexual violence or abuse in adulthood was derived from two questions about non-consensual sexual contact and forced sexual intercourse since age 16.

Standard demographic questions established sex (male, female), age (banded into 16–24, 25–34, 35–44, 45–54, 55 +) and marital status (single; married/cohabiting; separated/divorced/widowed). Ethnicity was self-ascribed, and, due to the relatively small number of participants in the White Other, Asian/Asian British, Black, Mixed and Other groups, grouped into White British and Other. Socioeconomic context was captured from participants’ housing tenure (owner-occupier, renting from a social landlord, renting from a private landlord) and employment status (employed, unemployed and looking for work, economically inactive). Household income was equivalised (to take account of number and ages of people living in the household), and analysed in quintiles. The questionnaire and further methodological details are published elsewhere [29].

### Statistical analysis

Analyses used weighted data and took account of the complex survey design (sample stratification and clustering) and non-response. Data management, descriptive and correlational analyses were conducted in SPSS v25 [35], with p-values and confidence intervals calculated at the 95% level. Stata v14.1 [36] was used for running multiple variable logistic regression analyses. Regression models were run for four binary-coded dependent variables: current CMD; past-year suicidal thoughts; past-year suicide attempts; and past-year non-suicidal self-harm. Each was analysed according to three sequential models. The first examined past-year exposure to threatening/obscene messages as an independent variable, controlling for potential demographic and socioeconomic confounders. The second model additionally controlled for exposure to forms of violence and abuse other than emotional abuse in adulthood; the final model additionally controlled for emotional abuse in adulthood as well. Multicollinearity was checked, and found not to be a problem, with the Variance Inflation Factor (VIF) for all variables below 2. Missing data were minimal for all variables and excluded from analyses, except for household income. Household income was not provided by 23.4% of the sample. It is likely that this was either because participants did not know the income of all household members or because income is considered sensitive to report. For this variable, therefore, those not providing a response were coded as ‘missing’ and retained in the analysis.

## Results

Our results suggest that in England around 96% (*n* = 6857) of adults aged 16 or over have had at least one partner or spouse. Men (5.4%, *n* = 122) were more likely than women (2.7%, *n* = 79) to have never had a partner (*p* < 0.001). Subsequent analyses are based on participants reporting at least one partner in their life, to avoid results being confounded by relationship history.

### Prevalence

One person in fifteen (6.6%; *n* = 484; 95% CI 5.9–7.3) had received two or more unwanted obscene or threatening texts, emails, letters, or cards, that had caused them fear, alarm or distress from a current or former partner. This was twice as likely in women (8.7%) as men (4.4%) (*p* < 0.001). 14.5% of women aged 16–24 reported receipt of threatening/obscene messages, three times the rate for men of the same age (4.3%). A quarter (25.1%) of people exposed to threatening/obscene messages had received more than one in the past year (1.7% of adults, *n* = 124). Two-fifths (40.1%) of those with past-year exposure had received such messages monthly or more often.

### Characteristics

Table 1 shows that people who had been exposed to threatening/obscene messages from a partner were more likely: to be young (aged 16–24 (18.4%) or 25–34 (28.7%), *p *= 0.005), single or divorced (52.7%, *p* < 0.001), unemployed (5.3%, *p* < 0.001), and living in rented accommodation (58.0%, *p* < 0.001) and lower income households (18.7%, *p* = 0.002). While associated with disadvantage, the experience of threatening/obscene messaging was evident in all groups. The profile of those exposed in the past year was similar to that for those who had ever been exposed, although recent experience was more likely in young. There was no significant association with ethnic group (*p* = 0.505), although the sample was too small to examine this definitively.

**Table 1:** Demographic and socioeconomic profile of those exposed and not exposed to repeated threatening/obscene messaging from a partner or ex-partner

		Total^a^		Past year exposure		Ever exposed (including past year)		Never exposed		*p*-value^b^
		*N*	Weighted %	*n*	Weighted %	*n*	Weighted %	*n*	Weighted %	
Total		6804	100	124	1.7	484	6.6	6320	93.4	
Characteristics										
Sex										
Men		2731	48.3	41	45.8	114	32.1	2617	49.4	< 0.001
Women		4073	51.7	83	54.2	369	67.9	3704	50.6	
Age										
16–24		461	12.5	18	24.3	53	18.4	408	12.1	0.005
25–34		971	17.5	43	33.0	129	28.7	842	16.7	
35–44		1099	16.6	27	17.8	106	20.0	993	16.4	
45–54		1213	18.2	25	19.5	110	20.0	1103	18.1	
55 +		3060	35.2	11	5.4	86	12.9	2974	36.8	
Ethnic group										
White British		5824	82.3	101	78.5	413	83.6	5411	82.2	0.505
Other		963	17.7	23	21.5	69	16.4	894	17.8	
Marital/cohabitation status										
Married/cohabiting		3896	64.6	25	29.7	181	47.3	3715	65.9	< 0.001
Single		1301	21.7	61	50.5	165	34.7	1136	20.7	
Divorced		1607	13.7	38	19.8	138	18.0	1469	13.4	
Economic activity										
Employed		3745	61.5	82	66.5	316	69.9	3429	60.9	< 0.001
Unemployed		189	3.1	7	6.1	23	5.3	166	2.9	
Other		2870	35.4	35	27.3	145	24.8	2725	36.2	
Tenure										
Owner occupied		4510	65.2	42	65.6	192	42.0	4318	66.8	< 0.001
Social renter		1084	15.0	30	14.8	134	26.0	950	14.2	
Private or other		1177	19.9	51	19.6	154	32.0	1023	19.0	
Equivalised household income quintiles										
Highest income		1072	16.4	11	7.5	50	11.0	1022	16.7	0.002
2nd		1037	15.2	19	13.7	73	16.6	964	15.2	
3rd		1151	15.8	28	21.3	88	16.7	1063	15.8	
4th		1145	15.0	23	20.9	84	15.6	1061	15.0	
Lowest income		1057	14.1	32	20.9	113	18.7	944	13.8	
Missing		1342	23.4	10	15.8	76	21.3	1266	23.6	

^a^Total: adults aged 16 or over reporting having had at least one partner (girlfriend/boyfriend/spouse)

^b^Association between characteristic and threatening or obscene messaging ever

People who received threatening/obscene messages from a current or former partner were more likely to have also experienced all the other types of violence and abuse examined. They were three times more likely than those who had not experienced such messaging to have been abused in childhood emotionally (30.2%, cf. 8.9%), sexually (23.5%, cf. 6.8%), and/or physically (26.5%, cf. 11.4%). They were also about five times more likely to have experienced emotional (59.4%, cf. 8.8%) or physical abuse from a partner (63.1%, cf. 11.4%), or sexual abuse (22.0%, cf. 4.4%) at some point in adulthood. Two-thirds (69.7%) of women and half of men (48.8%) who received threatening/obscene messages had experienced physical partner–violence at some point in adulthood, compared with 14.6% of women and 8.2% of men who had not received threatening/obscene messages.

Rates of CMD were more than twice as high in people who had received threatening/obscene messages (39.2%) than in those who had not (15.2%). This pattern was evident both in women (42.8%, cf. 18.3%) and men (31.5%, cf. 12.0%). Non-suicidal self-harm rates were higher in women (7.1%) and men (3.0%) exposed to such messages, than in women (1.7%) and men (1.3%) not exposed. Suicidal thoughts were three times more common in those exposed to messages (12.6%) than in those who were not (4.1%), while attempted suicide was four times more likely (2.0%, cf. 0.5%). Exposure to abusive messaging was also associated with harmful or dependent use of alcohol. For each of these outcomes, the pattern of association was similar for women and men (see Table 2).

**Table 2:** Prevalence of other types of violence and abuse, mental disorder, self-harm, suicidality and harmful/dependent alcohol use by whether exposed to threatening/obscene messages from a (ex)partner

	Men			Women			Total		
	Threatening/obscene messages	Not exposed	Total	Threatening/obscene messages	Not exposed	Total	Threatening/obscene messages	Not exposed	Total
Type of violence and abuse^a^	**%**	**%**	**%**	**%**	**%**	**%**	**%**	**%**	**%**
Physical abuse from caregiver in childhood	31.9	13.4	14.2	24.0	9.5	10.8	26.5	11.4	12.4
Sexual abuse in childhood	14.4	4.0	4.5	27.7	9.6	11.1	23.5	6.8	7.9
Emotional abuse from caregiver in childhood	33.8	7.7	8.8	28.6	10.1	11.7	30.2	8.9	10.3
Physical violence from partner in adulthood	48.8	8.2	10.0	69.7	14.6	19.3	63.1	11.4	14.8
Sexual abuse or violence in adulthood	6.8	1.5	1.7	29.1	7.3	9.2	22.0	4.4	5.6
Emotional abuse from partner in adulthood	42.1	4.9	6.6	67.6	12.7	17.4	59.4	8.8	12.2
Mental disorder and self-harm									
Common mental disorder (CMD)	31.5	12.0	12.9	42.8	18.3	20.5	39.2	15.2	16.8
Non-suicidal self-harm in past year	3.0	1.2	1.3	7.1	1.7	2.2	5.8	1.5	1.7
Suicidal thoughts in past year	13.0	4.4	4.8	12.4	3.8	4.6	12.6	4.1	4.7
Suicide attempt in past year	1.3	0.5	0.5	2.4	0.5	0.7	2.0	0.5	0.6
Harmful/dependent alcohol use	12.0	4.4	4.7	5.8	1.5	1.8	7.8	2.9	3.2
*N*	114	2617	2731	370	3703	4073	484	6320	6804

^a^CMD, suicidal thoughts, suicide attempt, non-suicidal self-harm and experience of all types of violence and abuse were more likely in those who had received threatening/obscene messages in the past year at *p* < 0.001

### Adjusted associations

In a model controlling for demographic and socioeconomic factors, people exposed to threatening/obscene messages in the past year had 3.95 times the odds of CMD compared with the rest of the population (95% CI 2.5–6.3, *p* < 0.001). After further adjustment for exposure to physical violence (in childhood and adulthood), sexual abuse (in childhood and adulthood), and emotional abuse (in childhood only), the association between recent threatening/obscene messages and CMD was attenuated but remained significant (adjusted OR [aOR] 2.20, 1.2–4.0, *p* = 0.011). Past-year messaging retained an independent association with CMD even when further controlling for experience of any form of emotional abuse from a partner at any point in adulthood (aOR 1.89, 1.0–3.5, *p* = 0.047), see Table 3 and supplementary materials.

**Table 3:** Threatening or obscene messages in past year as a risk factor for different mental health, self-harm, suicidality and dependence outcomes

	Models 1^a^				Models 2^b^				Models 3^c^			
	Adj. OR	Lower CI	Upper CI	*p*-value	Adj. OR	Lower CI	Upper CI	*p*-value	Adj. OR	Lower CI	Upper CI	*p*-value
Common mental disorder	3.95	2.48	6.31	< 0.001	2.20	1.20	4.05	0.011	1.89	1.01	3.55	0.047
Non-suicidal self-harm in past year	4.28	1.98	9.27	< 0.001	2.95	1.23	7.07	0.015	2.31	1.00	5.33	0.051
Suicidal thoughts in past year	3.76	2.13	6.63	0.000	2.19	1.13	4.24	0.021	2.00	1.06	3.78	0.034
Suicide attempt in past year	5.49	2.02	14.98	0.001	2.94	0.86	10.07	0.086	2.35	0.77	7.16	0.132
Harmful/dependent alcohol use	2.05	0.89	4.73	0.090	1.15	0.51	2.60	0.744	1.00	0.45	2.24	0.996

^a^Models 1: control for sex, age, marital status, tenure, equivalised household income, and threatening/obscene messaging in the past year

^b^Models 2: model 1 plus five indicators of violence and abuse; sexual and physical abuse in childhood or adulthood, and emotional abuse in childhood

^c^Models 3: model 2 plus emotional abuse in adulthood

Controlling for demographic and socioeconomic factors, those with past-year exposure to threatening/obscene messages had odds of non-suicidal self-harm in the past year 4.28 times higher than the rest of the population (2.0–9.3, *p* < 0.001). Further adjustment for physical violence and sexual abuse as a child and adult, as well as for childhood emotional abuse, reduced the odds ratio to 2.95 (1.2–7.1, *p* = 0.015). In the final model, which additionally adjusted for emotional abuse from a partner across adulthood, the effect size was attenuated (aOR 2.31), although the independent association between past-year threatening/obscene messages and non-suicidal self-harm was not significant at the 95% level (1.0–5.3, *p* = 0.051).

The odds of having suicidal thoughts in the past year remained 3.76 times higher in those exposed to messages than in those not exposed, when adjusting for demographic and socioeconomic factors (2.1–6.6, *p* < 0.001). In a model which additionally controlled for all other types of violence recorded in childhood and adulthood, threatening/obscene messages still had a significant independent association with suicidal thoughts (aOR 2.00, 1.1–3.8, *p* = 0.034).

People with past-year exposure to threatening/obscene messages had odds of attempted suicide in the past year 5.49 times higher than the rest of the population (2.0–15.0, *p* = 0.001) after adjustment for socioeconomic factors. Further adjustment for physical violence and sexual abuse in childhood and adulthood, as well as for childhood emotional abuse, reduced the odds ratio to 2.94 (0.9–10.1), which did not reach significance at the 95% level (*p* = 0.086). In the final model, additionally adjusted for any emotional abuse from a partner in adulthood, the effect size remained pronounced (aOR 2.35), although the independent association between messages and suicide attempts remained non-significant at the 95% level (0.8–7.2, *p* = 0.132).

After adjustment, exposure to abusive messaging in the past year was not associated with harmful or dependent use of alcohol (aOR 1.00, 0.45–2.24, *p* = 0.996).

## Discussion

Repeated threatening or obscene messaging causing fear, alarm or distress is a relatively common but rarely researched form of partner abuse experienced by one person in fifteen in England. Consistent with research on cyber IPV [37], those experiencing such messaging are more likely to be young, female, and living in socioeconomically disadvantaged circumstances, although it is also experienced by men, older people, and across socioeconomic groups.

Those experiencing threatening or obscene messaging are often also exposed to other forms of abuse and violence too. This cross sectional dataset provided a snapshot in time. We could not distinguish abusive messaging that presaged other forms of IPV (for example, as a feature of dating abuse) from that which occurred alongside other forms of IPV (perhaps to control a current partner when they are physically out of reach). The results are also consistent with research highlighting abusive messaging as a continuation of coercive control post-separation, after someone has left an abusive relationship [38]. This includes the use in ‘revenge porn’ after a relationship ends. While popular discourse around the non-consensual sharing of sexual images has focussed on the motivations of a ‘jilted sexual partner’ [39–41], this analysis refocuses attention on the harms to victims.

People who received repeated threatening or obscene messages from a current or previous partner in the past year were more likely to experience anxiety and depression, to engage in self-harm, and to be suicidal. These associations remained elevated even after adjusting for confounding for current socioeconomic disadvantage and exposure to a range of types of violence and abuse across the life course. Lindsay and colleagues [42] have shown how abusive messaging from an intimate partner increases the odds of experiencing fear as a result of victimisation, and that, in turn, fear was associated with an increased risk of depression. Our results are consistent with a trauma-informed understanding of mental health, and support the evidence base by demonstrating the additional effect that such communications can have. Health care professionals carrying out safety assessments should therefore enquire routinely and explicitly about messages and communications, and these should refer to any received from a previous as well as a current partner. Voluntary and statutory support services engaging with people who have left abusive relationships should establish whether survivors still have ongoing contact with former partners, and the nature of any communication, to ensure threats, intimidation, harassment and monitoring are not missed.

This study was unable to examine technology-enabled abuse in isolation. To respond to emerging risks—with implications for the frequency, immediacy, and visual content of threatening or obscene messaging—technology-enabled communications should be asked about in research and during safety assessments and accounted for during safety planning. New telephone numbers, contact details, and ideally devices, should be issued to those at risk [43]. Mobile providers and other suppliers should facilitate blocking other people across platforms. As technology may change the ways in which abuse is mediated, future surveys will need to adapt to ensure that the scale and impact of such experiences in adulthood and childhood is captured, including on longitudinal studies. The rapidity of such changes poses challenges for research, for service responses, and for victims.

This is the first analysis using representative English mental health survey data to present the national prevalence of threatening and obscene messaging from partners, together with its social and mental health correlates. The survey was based on a national probability sample and high-quality self-completion procedures in the context of a face to face interview. The identification of threatening or obscene messaging, however, was based on a single question, which did not distinguish between online and offline forms of communication, whether the perpetrator was a current or former partner, or whether messages were felt to be threatening or obscene. In the context of what Herman [44] calls ‘domestic terrorism’, however, sexual comments and pornographic images from a current or previous partner will often be experienced by women as *both* threatening and obscene. The question also asked only about the receipt of threatening or obscene messages that had actually provoked fear, distress, or alarm in the participant: in consequence, it failed to capture people who had received such messages but felt unaffected by them. The lower rate in men, for example, might have reflected the fact they were less likely to experience distress from such messages, or were less likely to disclose having felt distress. Recent (past year) experience was reported by relatively few (124) participants, and some of the analyses were accordingly underpowered. The findings, however, echo gendered dynamics identified in the context of the sending and receiving of sexualised images. Female teens receive far more unsolicited messages and images than males and are also more stigmatised for sending, for example, nude pictures in return [45]. The Conflict Tactics Scale (CTS) [34], which informed the questions used to capture other forms of partner violence and abuse, has been identified as problematic. The scale amalgamates non-equivalent types of gendered violence (for example, implying that being ‘slapped’ and ‘pinned down’ are equivalent) [46], and fails to capture context or impact [47].

The sampling frame excluded those living in residential or institutional settings or sleeping rough; and people living in domestic violence refuges and shelters were unlikely to have been selected. However, the proportion of the population not living in private households is small [48] and would have had little impact on the prevalence estimates and associations examined. While a survey response rate of 57% is in line with similar surveys, bias due to non-participation is inevitable. While cross sectional surveys using high quality general population samples are the best data source for prevalence and for profiling (understanding nuanced socioeconomic circumstances and the relationship with other types of violence and abuse), conclusions cannot be drawn about causality.

## Conclusion

This study supports earlier research in finding pronounced associations between emotional abuse and poor mental health and suicidality. When asking about violence and abuse, clinicians, law enforcement, and service providers need to ensure specific enquiry about threats, intimidation, and harassment from partners who may not be present in the home. Such enquiry, as well as future research, must address the potential for ongoing abuse from former partners, who may use remote communications to continue to exert threats and control [37]. Abusive messaging is one strategy of control that partners, especially former partners, use when their victim is not physically available [49]. In the context of the unintended impacts of ongoing physical distancing measures to mitigate against Covid-19 transmission [50–52], addressing abusive messaging has only gained in urgency.

Mental health services and future research should cover both online and offline modes of communication. Malicious communications in the context of IPV should feature in policy and legislation on both domestic violence and online harms. Our results support calls for technology companies to publish annual transparency reports to assess the prevalence of harmful content on their platforms. Hopefully through such means more data and evidence on online and offline facilitated forms of communication-enabled abuse will become available and will help to contextualise future research in this area.

### Supplementary Information

Below is the link to the electronic supplementary material.

Supplementary file1 (DOCX 107 kb)

## Data Availability

The corresponding author (SM) had full access to all the data in the study and had final responsibility for the decision to submit for publication. The APMS dataset is lodged with the UK Data Service archive. Permission to use the dataset for this analysis was obtained from NHS Digital. Requests for further use should be made to the Data Access Request Service at NHS Digital [https://digital.nhs.uk/services/data-access-request-service-dars].
