# Supplementary material for: Receiving threatening or obscene messages from a partner and mental health, self-harm and suicidality: results from the Adult Psychiatric Morbidity Survey
Source: Soc Psychiatry Psychiatr Epidemiol. 2021 Jul 28;58(12):1749–59. doi: 10.1007/s00127-021-02113-w (PMC8318057; doi:10.1007/s00127-021-02113-w)
Supplement: Supplementary file 1 — Supplementary file1 (DOCX 107 kb) [file 127_2021_2113_MOESM1_ESM.docx]

**Receiving threatening or obscene messages from a partner**

**Web annex: Supplementary materials**

**Table S1 Adjusted odds ratios for common mental disorder (CMD)**

|  | **Model 1**^a^ (n=6,740) | | | | **Model 2^b^** (n=6,676) | | | | **Model 3^c^** (n=6,652) | | | |
| --- | --- | --- | --- | --- | --- | --- | --- | --- | --- | --- | --- | --- |
|  | **Adj.**  **OR** | **Lower CI** | **Upper CI** | **p-**  **value** | **Adj. OR** | **Lower CI** | **Upper CI** | **p-value** | **Adj.**  **OR** | **Lower CI** | **Upper CI** | **p-**  **value** |
| **Threatening/obscene messaging in past year** | **3.95** | **2.478** | **6.308** | **<0.001** | **2.20** | **1.200** | **4.045** | **0.011** | **1.89** | **1.009** | **3.548** | **0.047** |
| **Sex** |  |  |  |  |  |  |  |  |  |  |  |  |
| Male | Ref |  |  |  | Ref |  |  |  | Ref |  |  |  |
| Female | 1.68 | 1.428 | 1.975 | <0.001 | 1.41 | 1.183 | 1.673 | <0.001 | 1.34 | 1.125 | 1.596 | 0.001 |
| **Age group** |  |  |  |  |  |  |  |  |  |  |  |  |
| 16-24 | Ref |  |  |  | Ref |  |  |  | Ref |  |  |  |
| 25-34 | 1.25 | 0.916 | 1.718 | 0.157 | 1.26 | 0.912 | 1.729 | 0.162 | 1.22 | 0.884 | 1.691 | 0.225 |
| 35-44 | 1.37 | 0.983 | 1.923 | 0.063 | 1.20 | 0.848 | 1.687 | 0.307 | 1.14 | 0.803 | 1.607 | 0.471 |
| 45-54 | 1.44 | 1.032 | 2.003 | 0.032 | 1.27 | 0.899 | 1.801 | 0.173 | 1.21 | 0.853 | 1.725 | 0.282 |
| 55+ | 0.90 | 0.646 | 1.254 | 0.531 | 0.89 | 0.637 | 1.238 | 0.482 | 0.86 | 0.618 | 1.206 | 0.388 |
| **Marital status** |  |  |  |  |  |  |  |  |  |  |  |  |
| Married/cohabitating | Ref |  |  |  | Ref |  |  |  | Ref |  |  |  |
| Single | 1.36 | 1.102 | 1.682 | 0.004 | 1.34 | 1.081 | 1.668 | 0.008 | 1.29 | 1.035 | 1.615 | 0.024 |
| Divorced/separated/ widowed | 1.08 | 0.895 | 1.291 | 0.437 | 0.99 | 0.822 | 1.197 | 0.934 | 0.93 | 0.769 | 1.120 | 0.436 |
| **Tenure** |  |  |  |  |  |  |  |  |  |  |  |  |
| Owner occupied | Ref |  |  |  | Ref |  |  |  | Ref |  |  |  |
| Social renter | 1.99 | 1.629 | 2.435 | <0.001 | 1.68 | 1.357 | 2.086 | <0.001 | 1.60 | 1.286 | 1.994 | <0.001 |
| Private renter | 1.27 | 1.038 | 1.561 | 0.021 | 1.06 | 0.849 | 1.316 | 0.619 | 1.03 | 0.827 | 1.283 | 0.794 |
| **Equivalised household income** |  |  |  |  |  |  |  |  |  |  |  |  |
| Highest income | Ref |  |  |  | Ref |  |  |  | Ref |  |  |  |
| 2^nd^ | 1.01 | 0.755 | 1.346 | 0.956 | 1.00 | 0.744 | 1.347 | 0.993 | 0.99 | 0.736 | 1.339 | 0.961 |
| 3^rd^ | 1.28 | 0.968 | 1.681 | 0.083 | 1.35 | 1.015 | 1.796 | 0.039 | 1.30 | 0.974 | 1.741 | 0.075 |
| 4^th^ | 1.28 | 0.969 | 1.698 | 0.082 | 1.33 | 0.988 | 1.790 | 0.060 | 1.30 | 0.960 | 1.752 | 0.090 |
| Lowest income | 1.97 | 1.503 | 2.583 | <0.001 | 1.96 | 1.467 | 2.615 | <0.001 | 1.89 | 1.404 | 2.543 | <0.001 |
| Missing | 1.28 | 0.967 | 1.698 | 0.084 | 1.39 | 1.040 | 1.865 | 0.027 | 1.38 | 1.028 | 1.862 | 0.032 |
| **Violence and abuse across the life course** |  |  |  |  |  |  |  |  |  |  |  |  |
| Sexual abuse in childhood |  |  |  |  | 1.63 | 1.267 | 2.088 | <0.001 | 1.55 | 1.196 | 2.007 | 0.001 |
| Physical abuse from caregiver in childhood |  |  |  |  | 1.23 | 0.972 | 1.564 | 0.084 | 1.22 | 0.956 | 1.556 | 0.110 |
| Emotional abuse from caregiver in childhood |  |  |  |  | 2.64 | 2.089 | 3.346 | <0.001 | 2.55 | 1.999 | 3.241 | <0.001 |
| Sexual abuse in adulthood |  |  |  |  | 1.55 | 1.172 | 2.044 | 0.002 | 1.40 | 1.054 | 1.869 | 0.020 |
| Physical violence from (ex)partner in adulthood |  |  |  |  | 1.94 | 1.611 | 2.336 | <0.001 | 1.38 | 1.096 | 1.736 | 0.006 |
| Emotional abuse from (ex)partner in adulthood |  |  |  |  |  |  |  |  | 2.19 | 1.740 | 2.748 | <0.001 |

^a^ Model 1: controlling for sex, age, marital status, tenure, equivalised household income, and threatening/ obscene messaging in the past year. ^b^ Model 2: model 1 plus five indicators of violence and abuse ever; sexual and physical abuse in childhood or adulthood, and emotional abuse in childhood. ^c^ Model 3: model 2 plus emotional abuse in adulthood. In a further model (not shown) which additionally adjusts for victims’ harmful or dependent alcohol use, the OR for threatening/obscene messaging remains essentially unchanged (1.91).

**Table S2 Adjusted odds ratios for non-suicidal self-harm in the past year**

|  | **Model 1**^a^ (n=6,735) | | | | **Model 2^b^** (n=6,671) | | | | **Model 3^c^** (n=6,648) | | | |
| --- | --- | --- | --- | --- | --- | --- | --- | --- | --- | --- | --- | --- |
|  | **Adj.**  **OR** | **Lower CI** | **Upper CI** | **p-**  **value** | **Adj. OR** | **Lower CI** | **Upper CI** | **p-value** | **Adj.**  **OR** | **Lower CI** | **Upper CI** | **p-**  **value** |
| **Threatening/obscene messaging in past year** | **4.28** | **1.980** | **9.272** | **<0.001** | **2.95** | **1.231** | **7.069** | **0.015** | **2.31** | **0.998** | **5.332** | **0.051** |
| **Sex** |  |  |  |  |  |  |  |  |  |  |  |  |
| Male | Ref |  |  |  | Ref |  |  |  | Ref |  |  |  |
| Female | 1.60 | 0.939 | 2.735 | 0.084 | 1.38 | 0.763 | 2.504 | 0.285 | 1.27 | 0.688 | 2.331 | 0.447 |
| **Age group** |  |  |  |  |  |  |  |  |  |  |  |  |
| 16-24 | Ref |  |  |  | Ref |  |  |  | Ref |  |  |  |
| 25-34 | 0.34 | 0.172 | 0.664 | 0.002 | 0.27 | 0.133 | 0.556 | <0.001 | 0.27 | 0.130 | 0.545 | <0.001 |
| 35-44 | 0.27 | 0.129 | 0.564 | 0.001 | 0.19 | 0.086 | 0.425 | <0.001 | 0.18 | 0.082 | 0.404 | <0.001 |
| 45-54 | 0.25 | 0.110 | 0.558 | 0.001 | 0.18 | 0.075 | 0.418 | <0.001 | 0.16 | 0.065 | 0.373 | <0.001 |
| 55+ | 0.06 | 0.023 | 0.147 | <0.001 | 0.05 | 0.020 | 0.132 | <0.001 | 0.05 | 0.018 | 0.124 | <0.001 |
| **Marital status** |  |  |  |  |  |  |  |  |  |  |  |  |
| Married/cohabitating | Ref |  |  |  | Ref |  |  |  | Ref |  |  |  |
| Single | 3.26 | 1.703 | 6.248 | <0.001 | 3.01 | 1.519 | 5.948 | 0.002 | 2.94 | 1.468 | 5.907 | 0.002 |
| Divorced/separated/ widowed | 2.91 | 1.492 | 5.689 | 0.002 | 2.65 | 1.339 | 5.257 | 0.005 | 2.47 | 1.244 | 4.902 | 0.010 |
| **Tenure** |  |  |  |  |  |  |  |  |  |  |  |  |
| Owner occupied | Ref |  |  |  | Ref |  |  |  | Ref |  |  |  |
| Social renter | 2.82 | 1.446 | 5.518 | 0.002 | 2.03 | 0.986 | 4.169 | 0.054 | 1.94 | 0.933 | 4.044 | 0.076 |
| Private renter | 2.30 | 1.244 | 4.266 | 0.008 | 1.77 | 0.942 | 3.330 | 0.076 | 1.76 | 0.939 | 3.291 | 0.078 |
| **Equivalised household income** |  |  |  |  |  |  |  |  |  |  |  |  |
| Highest income | Ref |  |  |  | Ref |  |  |  | Ref |  |  |  |
| 2^nd^ | 1.93 | 0.458 | 8.104 | 0.370 | 2.40 | 0.500 | 11.546 | 0.273 | 2.37 | 0.519 | 10.796 | 0.265 |
| 3^rd^ | 2.62 | 0.729 | 9.412 | 0.140 | 3.37 | 0.836 | 13.563 | 0.088 | 3.12 | 0.809 | 12.033 | 0.098 |
| 4^th^ | 1.66 | 0.469 | 5.866 | 0.431 | 1.71 | 0.408 | 7.167 | 0.463 | 1.52 | 0.382 | 6.045 | 0.552 |
| Lowest income | 2.98 | 0.868 | 10.217 | 0.082 | 3.33 | 0.823 | 13.443 | 0.091 | 3.06 | 0.793 | 11.817 | 0.104 |
| Missing | 1.49 | 0.404 | 5.509 | 0.548 | 2.00 | 0.472 | 8.502 | 0.346 | 1.89 | 0.474 | 7.536 | 0.366 |
| **Violence and abuse across the life course** |  |  |  |  |  |  |  |  |  |  |  |  |
| Sexual abuse in childhood |  |  |  |  | 1.32 | 0.683 | 2.544 | 0.410 | 1.15 | 0.594 | 2.228 | 0.678 |
| Physical abuse from caregiver in childhood |  |  |  |  | 1.13 | 0.602 | 2.139 | 0.695 | 1.10 | 0.588 | 2.043 | 0.772 |
| Emotional abuse from caregiver in childhood |  |  |  |  | 7.63 | 3.847 | 15.145 | <0.001 | 7.63 | 3.867 | 15.036 | <0.001 |
| Sexual abuse in adulthood |  |  |  |  | 2.34 | 1.106 | 4.970 | 0.026 | 2.18 | 1.025 | 4.652 | 0.043 |
| Physical violence from a partner in adulthood |  |  |  |  | 0.81 | 0.394 | 1.650 | 0.555 | 0.50 | 0.200 | 1.229 | 0.130 |
| Emotional abuse from a partner in adulthood |  |  |  |  |  |  |  |  | 2.84 | 1.212 | 6.653 | 0.016 |

^a^ Model 1: controlling for sex, age, marital status, tenure, equivalised household income, and threatening/ obscene messaging in the past year. ^b^ Model 2: model 1 plus five indicators of violence and abuse; sexual and physical abuse in childhood or adulthood, and emotional abuse in childhood. ^c^ Model 3: model 2 plus emotional abuse in adulthood. In a further model (not shown) which additionally adjusts for victims’ harmful or dependent alcohol use, the OR for threatening/obscene messaging attenuated slightly (2.23).

**Table S3 Adjusted odds ratios for suicidal thoughts in the past year**

|  | **Model 1**^a^ (n=6,740) | | | | **Model 2^b^** (n=6,676) | | | | **Model 3^c^** (n=6,523) | | | |
| --- | --- | --- | --- | --- | --- | --- | --- | --- | --- | --- | --- | --- |
|  | **Adj.**  **OR** | **Lower CI** | **Upper CI** | **p-**  **value** | **Adj. OR** | **Lower CI** | **Upper CI** | **p-value** | **Adj.**  **OR** | **Lower CI** | **Upper CI** | **p-**  **value** |
| **Threatening/obscene messaging in past year** | **3.76** | **2.126** | **6.632** | **0.000** | **2.19** | **1.125** | **4.242** | **0.021** | **2.00** | **1.056** | **3.775** | **0.034** |
| **Sex** |  |  |  |  |  |  |  |  |  |  |  |  |
| Male | Ref |  |  |  | Ref |  |  |  | Ref |  |  |  |
| Female | 0.88 | 0.669 | 1.161 | 0.368 | 0.73 | 0.548 | 0.969 | 0.029 | 0.70 | 0.526 | 0.943 | 0.019 |
| **Age group** |  |  |  |  |  |  |  |  |  |  |  |  |
| 16-24 | Ref |  |  |  | Ref |  |  |  | Ref |  |  |  |
| 25-34 | 0.85 | 0.518 | 1.400 | 0.525 | 0.83 | 0.495 | 1.391 | 0.478 | 0.82 | 0.486 | 1.374 | 0.445 |
| 35-44 | 0.84 | 0.512 | 1.363 | 0.470 | 0.72 | 0.428 | 1.203 | 0.208 | 0.69 | 0.409 | 1.154 | 0.156 |
| 45-54 | 0.99 | 0.595 | 1.656 | 0.978 | 0.88 | 0.516 | 1.507 | 0.644 | 0.84 | 0.488 | 1.434 | 0.515 |
| 55+ | 0.52 | 0.311 | 0.875 | 0.014 | 0.51 | 0.304 | 0.856 | 0.011 | 0.50 | 0.294 | 0.834 | 0.008 |
| **Marital status** |  |  |  |  |  |  |  |  |  |  |  |  |
| Married/cohabitating | Ref |  |  |  | Ref |  |  |  | Ref |  |  |  |
| Single | 2.12 | 1.427 | 3.141 | <0.001 | 2.01 | 1.341 | 3.028 | 0.001 | 1.94 | 1.281 | 2.933 | 0.002 |
| Divorced/separated/ widowed | 2.11 | 1.543 | 2.876 | <0.001 | 1.98 | 1.447 | 2.702 | <0.001 | 1.92 | 1.400 | 2.628 | <0.001 |
| **Tenure** |  |  |  |  |  |  |  |  |  |  |  |  |
| Owner occupied | Ref |  |  |  | Ref |  |  |  | Ref |  |  |  |
| Social renter | 1.86 | 1.283 | 2.689 | 0.001 | 1.53 | 1.049 | 2.241 | 0.027 | 1.49 | 1.015 | 2.188 | 0.042 |
| Private renter | 1.30 | 0.899 | 1.870 | 0.164 | 1.08 | 0.736 | 1.596 | 0.683 | 1.08 | 0.734 | 1.590 | 0.696 |
| **Equivalised household income** |  |  |  |  |  |  |  |  |  |  |  |  |
| Highest income | Ref |  |  |  |  |  |  |  | Ref |  |  |  |
| 2^nd^ | 0.63 | 0.336 | 1.182 | 0.150 | 0.63 | 0.339 | 1.180 | 0.149 | 0.63 | 0.338 | 1.180 | 0.149 |
| 3^rd^ | 0.89 | 0.527 | 1.511 | 0.672 | 0.90 | 0.522 | 1.558 | 0.709 | 0.87 | 0.505 | 1.515 | 0.632 |
| 4^th^ | 0.93 | 0.580 | 1.499 | 0.771 | 0.95 | 0.576 | 1.567 | 0.840 | 0.93 | 0.561 | 1.532 | 0.768 |
| Lowest income | 1.27 | 0.831 | 1.929 | 0.271 | 1.20 | 0.773 | 1.851 | 0.419 | 1.16 | 0.749 | 1.806 | 0.499 |
| Missing | 0.75 | 0.452 | 1.240 | 0.260 | 0.83 | 0.500 | 1.388 | 0.483 | 0.82 | 0.493 | 1.376 | 0.458 |
| **Violence and abuse across the life course** |  |  |  |  |  |  |  |  |  |  |  |  |
| Sexual abuse in childhood |  |  |  |  | 0.94 | 0.620 | 1.431 | 0.779 | 0.89 | 0.589 | 1.360 | 0.602 |
| Physical abuse from caregiver in childhood |  |  |  |  | 1.21 | 0.804 | 1.810 | 0.364 | 1.18 | 0.783 | 1.769 | 0.432 |
| Emotional abuse from caregiver in childhood |  |  |  |  | 2.76 | 1.822 | 4.184 | <0.001 | 2.74 | 1.804 | 4.148 | <0.001 |
| Sexual abuse in adulthood |  |  |  |  | 2.01 | 1.320 | 3.072 | 0.001 | 1.92 | 1.257 | 2.929 | 0.003 |
| Physical violence from a partner in adulthood |  |  |  |  | 1.56 | 1.095 | 2.224 | 0.014 | 1.26 | 0.820 | 1.926 | 0.293 |
| Emotional abuse from a partner in adulthood |  |  |  |  |  |  |  |  | 1.61 | 1.089 | 2.393 | 0.017 |

^a^ Model 1: controlling for sex, age, marital status, tenure, equivalised household income, and threatening/ obscene messaging in the past year. ^b^ Model 2: model 1 plus five indicators of violence and abuse; sexual and physical abuse in childhood or adulthood, and emotional abuse in childhood. ^c^ Model 3: model 2 plus emotional abuse in adulthood. In a further model (not shown) which additionally adjusts for victims’ harmful or dependent alcohol use, the OR for threatening/obscene messaging attenuated slightly (1.96).

**Table S4. Adjusted odds ratios for suicide attempt in the past year**

|  | **Model 1**^a^ (n=6,731) | | | | **Model 2^b^** (n=6,678) | | | | **Model 3^c^** (n=6,644) | | | |
| --- | --- | --- | --- | --- | --- | --- | --- | --- | --- | --- | --- | --- |
|  | **Adj.**  **OR** | **Lower CI** | **Upper CI** | **p-**  **value** | **Adj. OR** | **Lower CI** | **Upper CI** | **p-value** | **Adj.**  **OR** | **Lower CI** | **Upper CI** | **p-**  **value** |
| **Threatening/obscene messaging in past year** | **5.49** | **2.015** | **14.984** | **0.001** | **2.94** | **0.856** | **10.066** | **0.086** | **2.35** | **0.772** | **7.161** | **0.132** |
| **Sex** |  |  |  |  |  |  |  |  |  |  |  |  |
| Male | Ref |  |  |  | Ref |  |  |  | Ref |  |  |  |
| Female | 0.81 | 0.423 | 1.543 | 0.517 | 0.63 | 0.309 | 1.284 | 0.203 | 0.55 | 0.265 | 1.158 | 0.116 |
| **Age group** |  |  |  |  |  |  |  |  |  |  |  |  |
| 16-24 | Ref |  |  |  | Ref |  |  |  | Ref |  |  |  |
| 25-34 | 0.35 | 0.141 | 0.870 | 0.024 | 0.27 | 0.101 | 0.735 | 0.010 | 0.27 | 0.104 | 0.722 | 0.009 |
| 35-44 | 0.51 | 0.212 | 1.240 | 0.138 | 0.34 | 0.138 | 0.820 | 0.017 | 0.32 | 0.134 | 0.766 | 0.011 |
| 45-54 | 0.25 | 0.086 | 0.696 | 0.008 | 0.13 | 0.042 | 0.413 | 0.001 | 0.11 | 0.035 | 0.381 | <0.001 |
| 55+ | 0.15 | 0.056 | 0.381 | <0.001 | 0.11 | 0.043 | 0.299 | <0.001 | 0.10 | 0.039 | 0.280 | <0.001 |
| **Marital status** |  |  |  |  |  |  |  |  |  |  |  |  |
| Married/cohabitating | Ref |  |  |  | Ref |  |  |  | Ref |  |  |  |
| Single | 3.14 | 1.204 | 8.174 | 0.019 | 2.84 | 1.074 | 7.526 | 0.035 | 2.78 | 1.032 | 7.501 | 0.043 |
| Divorced/separated/ widowed | 3.33 | 1.659 | 6.669 | 0.001 | 3.41 | 1.694 | 6.864 | 0.001 | 3.20 | 1.563 | 6.558 | 0.002 |
| **Tenure** |  |  |  |  |  |  |  |  |  |  |  |  |
| Owner occupied | Ref |  |  |  | Ref |  |  |  | Ref |  |  |  |
| Social renter | 4.12 | 1.611 | 10.519 | 0.003 | 2.88 | 1.116 | 7.430 | 0.029 | 2.69 | 1.035 | 6.986 | 0.042 |
| Private renter | 1.59 | 0.598 | 4.243 | 0.351 | 1.04 | 0.372 | 2.935 | 0.934 | 1.01 | 0.363 | 2.783 | 0.992 |
| **Equivalised household income** |  |  |  |  |  |  |  |  |  |  |  |  |
| Highest income | Ref |  |  |  | Ref |  |  |  | Ref |  |  |  |
| 2^nd^ | 2.87 | 0.351 | 23.425 | 0.325 | 2.87 | 0.355 | 23.251 | 0.322 | 2.79 | 0.339 | 22.932 | 0.339 |
| 3^rd^ | 4.08 | 0.695 | 23.918 | 0.119 | 4.55 | 0.777 | 26.605 | 0.093 | 4.08 | 0.691 | 24.036 | 0.120 |
| 4^th^ | 3.83 | 0.726 | 20.204 | 0.113 | 3.35 | 0.600 | 18.669 | 0.168 | 3.00 | 0.556 | 16.219 | 0.201 |
| Lowest income | 5.67 | 1.118 | 28.772 | 0.036 | 4.75 | 0.896 | 25.140 | 0.067 | 4.33 | 0.824 | 22.762 | 0.083 |
| Missing | 5.00 | 0.923 | 27.038 | 0.062 | 6.24 | 1.136 | 34.235 | 0.035 | 5.87 | 1.064 | 32.348 | 0.042 |
| **Violence and abuse across the life course** |  |  |  |  |  |  |  |  |  |  |  |  |
| Sexual abuse in childhood |  |  |  |  | 2.32 | 0.919 | 5.851 | 0.075 | 2.03 | 0.854 | 4.829 | 0.108 |
| Physical abuse from caregiver in childhood |  |  |  |  | 2.12 | 1.000 | 4.505 | 0.050 | 2.04 | 0.956 | 4.366 | 0.065 |
| Emotional abuse from caregiver in childhood |  |  |  |  | 3.73 | 1.621 | 8.572 | 0.002 | 3.64 | 1.619 | 8.169 | 0.002 |
| Sexual abuse in adulthood |  |  |  |  | 1.33 | 0.550 | 3.216 | 0.526 | 1.23 | 0.536 | 2.840 | 0.620 |
| Physical violence from a partner in adulthood |  |  |  |  | 1.26 | 0.525 | 3.033 | 0.603 | 0.79 | 0.277 | 2.243 | 0.655 |
| Emotional abuse from a partner in adulthood |  |  |  |  |  |  |  |  | 2.92 | 1.180 | 7.245 | 0.021 |

^a^ Model 1: controlling for sex, age, marital status, tenure, equivalised household income, and threatening/ obscene messaging in the past year. ^b^ Model 2: model 1 plus five indicators of violence and abuse; sexual and physical abuse in childhood or adulthood, and emotional abuse in childhood. ^c^ Model 3: model 2 plus emotional abuse in adulthood. In a further model (not shown) which additionally adjusts for victims’ harmful or dependent alcohol use, the OR for threatening/obscene messaging attenuated slightly (2.25).

**Table S5. Adjusted odds ratios for harmful/dependent alcohol use (AUDIT 16+)**

|  | **Model 1**^a^ (n=6,729) | | | | **Model 2^b^** (n=6,665) | | | | **Model 3^c^** (n=6,642) | | | |
| --- | --- | --- | --- | --- | --- | --- | --- | --- | --- | --- | --- | --- |
|  | **Adj.**  **OR** | **Lower CI** | **Upper CI** | **p-**  **value** | **Adj. OR** | **Lower CI** | **Upper CI** | **p-value** | **Adj.**  **OR** | **Lower CI** | **Upper CI** | **p-**  **value** |
| **Threatening/obscene messaging in past year** | **2.05** | **0.893** | **4.728** | **0.090** | **1.15** | **0.506** | **2.595** | **0.744** | **1.00** | **0.445** | **2.237** | **0.996** |
| **Sex** |  |  |  |  |  |  |  |  |  |  |  |  |
| Male | Ref |  |  |  | Ref |  |  |  | Ref |  |  |  |
| Female | 0.37 | 0.260 | 0.533 | 0 | 0.28 | 0.195 | 0.414 | 0.000 | 0.27 | 0.182 | 0.391 | 0.000 |
| **Age group** |  |  |  |  |  |  |  |  |  |  |  |  |
| 16-24 | Ref |  |  |  | Ref |  |  |  | Ref |  |  |  |
| 25-34 | 1.16 | 0.621 | 2.180 | 0.635 | 1.03 | 0.551 | 1.915 | 0.932 | 1.02 | 0.543 | 1.903 | 0.959 |
| 35-44 | 1.50 | 0.807 | 2.806 | 0.198 | 1.25 | 0.665 | 2.344 | 0.488 | 1.20 | 0.636 | 2.271 | 0.571 |
| 45-54 | 1.06 | 0.544 | 2.056 | 0.869 | 0.88 | 0.454 | 1.713 | 0.710 | 0.85 | 0.434 | 1.665 | 0.635 |
| 55+ | 0.71 | 0.368 | 1.364 | 0.302 | 0.64 | 0.331 | 1.227 | 0.177 | 0.62 | 0.322 | 1.192 | 0.151 |
| **Marital status** |  |  |  |  |  |  |  |  |  |  |  |  |
| Married/cohabitating | Ref |  |  |  | Ref |  |  |  | Ref |  |  |  |
| Single | 2.61 | 1.775 | 3.830 | 0 | 2.52 | 1.715 | 3.692 | 0.000 | 2.46 | 1.678 | 3.608 | 0.000 |
| Divorced/separated/ widowed | 1.17 | 0.700 | 1.972 | 0.542 | 1.09 | 0.638 | 1.851 | 0.760 | 1.04 | 0.608 | 1.787 | 0.878 |
| **Tenure** |  |  |  |  |  |  |  |  |  |  |  |  |
| Owner occupied | Ref |  |  |  | Ref |  |  |  | Ref |  |  |  |
| Social renter | 1.49 | 0.912 | 2.450 | 0.110 | 1.17 | 0.562 | 0.686 | 1.997 | 1.12 | 0.664 | 1.901 | 0.664 |
| Private renter | 1.44 | 0.925 | 2.250 | 0.106 | 1.26 | 0.336 | 0.785 | 2.030 | 1.23 | 0.764 | 1.984 | 0.391 |
| **Equivalised household income** |  |  |  |  |  |  |  |  |  |  |  |  |
| Highest income | Ref |  |  |  | Ref |  |  |  | Ref |  |  |  |
| 2^nd^ | 0.85 | 0.512 | 1.424 | 0.544 | 0.84 | 0.505 | 0.508 | 1.397 | 0.84 | 0.506 | 1.391 | 0.495 |
| 3^rd^ | 0.77 | 0.413 | 1.438 | 0.412 | 0.82 | 0.528 | 0.434 | 1.535 | 0.79 | 0.419 | 1.505 | 0.479 |
| 4^th^ | 0.65 | 0.348 | 1.225 | 0.184 | 0.69 | 0.262 | 0.358 | 1.324 | 0.67 | 0.346 | 1.293 | 0.231 |
| Lowest income | 0.89 | 0.480 | 1.657 | 0.718 | 0.85 | 0.636 | 0.443 | 1.646 | 0.82 | 0.421 | 1.591 | 0.554 |
| Missing | 0.68 | 0.369 | 1.240 | 0.205 | 0.73 | 0.322 | 0.386 | 1.368 | 0.73 | 0.388 | 1.382 | 0.335 |
| **Violence and abuse across the life course** |  |  |  |  |  |  |  |  |  |  |  |  |
| Sexual abuse in childhood |  |  |  |  | 2.05 | 0.009 | 0.506 | 2.595 | 1.92 | 1.122 | 3.287 | 0.018 |
| Physical abuse from caregiver in childhood |  |  |  |  | 1.12 | 0.646 | 1.202 | 3.506 | 1.10 | 0.674 | 1.782 | 0.710 |
| Emotional abuse from caregiver in childhood |  |  |  |  | 1.44 | 0.224 | 0.688 | 1.825 | 1.40 | 0.783 | 2.499 | 0.256 |
| Sexual abuse in adulthood |  |  |  |  | 1.73 | 0.059 | 0.801 | 2.577 | 1.60 | 0.895 | 2.872 | 0.112 |
| Physical violence from a partner in adulthood |  |  |  |  | 2.03 | 0.001 | 0.979 | 3.051 | 1.60 | 0.973 | 2.616 | 0.064 |
| Emotional abuse from a partner in adulthood |  |  |  |  |  |  |  |  | 1.90 | 1.059 | 3.392 | 0.031 |

^a^ Model 1: controlling for sex, age, marital status, tenure, equivalised household income, and threatening/obscene messaging in the past year. ^b^ Model 2: model 1 plus five indicators of violence and abuse; sexual and physical abuse in childhood or adulthood, and emotional abuse in childhood. ^c^ Model 3: model 2 plus emotional abuse in adulthood.
